# Recurrent Pregnancy-Related Pure Red Cell Aplasia Responsive to Combined Corticosteroid and Azathioprine Therapy: A Management Dilemma

**DOI:** 10.4274/tjh.galenos.2020.2020.0170

**Published:** 2020-08-28

**Authors:** Ashwin Rao, Rashmi Rao

**Affiliations:** 1 Salem Polyclinic, Department of Obstetrics and Gynecology, Tamil Nadu, India

**Keywords:** PRCA, Acquired pure red cell aplasia, Anemia in pregnancy, Hypoproliferation, Erythroid progenitors, Promegaloblast

## To the Editor,

Pure red cell aplasia (PRCA) is associated with normocytic normochromic anemia, severe reticulocytopenia, and reduced erythroid precursors in the bone marrow [1]. PRCA may be congenital or acquired. Among the acquired cases, pregnancy has been associated with PRCA [2].

**Figure 1:**
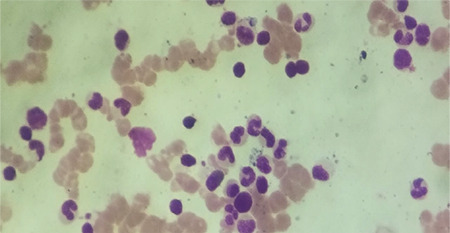
Bone marrow aspirate cytology showed occasional basophilic erythroblasts and promegaloblasts with moderate lymphocytosis.

**Figure 2:**
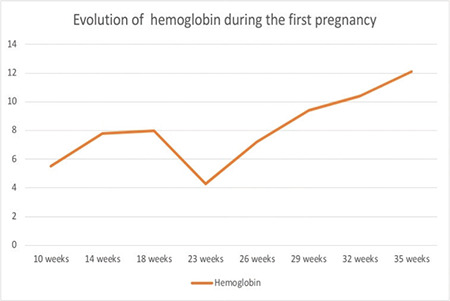
Hemoglobin values during first pregnancy.

A 25-year-old primigravid patient presented at 10 weeks of gestation with severe anemia with breathlessness for 2 months with no history of fever, jaundice, or weight loss. On examination, she had pallor but no icterus, peripheral lymphadenopathy, or hepatosplenomegaly. Laboratory investigations showed a hemoglobin level of 5.5 g/dL, red blood cell (RBC) count of 1.53 million/mm^3^, and white blood cell (WBC) count of 5280/µL. Her mean corpuscular volume, mean corpuscular hemoglobin, and mean corpuscular hemoglobin concentration were 81.2 fL, 25.8 pg, and 31.8 g/dL, respectively. A peripheral smear showed normocytic normochromic RBCs with occasional spherocytes with reticulocytes of 0.3%. Hence, the absolute reticulocyte count was 200/µL and the reticulocyte index was 0.15, which was suggestive of hypoproliferation. Ultrasound showed a single live intrauterine pregnancy corresponding to 9 weeks. On admission, packed RBCs were transfused. Hemoglobin electrophoresis was performed, which ruled out thalassemia. Other investigations for liver, renal, and endocrine functions were normal. Bone marrow aspirate cytology (Figure 1) showed occasional basophilic erythroblasts and promegaloblasts with moderate lymphocytosis. The percentage of erythroid precursors was 0.8% and the myeloid erythroid ratio was 85:1. The overall marrow cellularity was near normal. Serological tests for toxoplasma, rubella, cytomegalovirus, hepatitis B and C antibodies, and parvovirus IgM were found to be negative. Tests for antinuclear antibodies (ANA), ds-DNA, anticardiolipin antibodies, and lupus anticoagulant were also negative. The patient was started on oral prednisolone at 1 mg/kg body weight daily and was slowly tapered to 5 mg/day in the second trimester. At 23 weeks, her hemoglobin was 4.3 g/dL (Figure 2), although there was no history of bleeding; hence, oral azathioprine at 50 mg twice daily was started. Hemoglobin levels were checked periodically and corrected. The fetal growth and development were assessed regularly. Subsequently, her hemoglobin improved and she did not require further blood transfusions. At 38 weeks of gestation, she vaginally delivered a male baby weighing 2.6 kg. There were no congenital abnormalities observed. After delivery, hemoglobin was found to be 11.2 g/dL. The steroids were tapered and stopped after 4 weeks. She was in remission for 2 years when she presented with amenorrhea for 2 months associated with breathlessness. Ultrasound revealed intrauterine gestation corresponding to 8 weeks. Her hemoglobin was 4.3 g/dL with hematological findings as observed previously. Oral prednisolone was started and packed RBCs were transfused. As she was unwilling to continue the pregnancy, medical termination of pregnancy was performed, a copper intrauterine device was inserted, and prednisolone was tapered as before.

PRCA rarely occurs during pregnancy but it should be considered as a cause of refractory anemia in pregnancy. It can manifest in any trimester and is diagnosed by bone marrow aspirate cytology. In pregnancy, management is performed with prednisolone, although immunosuppressive therapy has also been used [2]. In Table 1, the different modes of treatment used in cases of pregnancy-related PRCA by different authors are summarized [3,4,5,6,7]. PRCA in pregnancy has a better prognosis compared to pre-existing PRCA and aplastic anemia in pregnancy. In general, PRCA that develops during pregnancy spontaneously resolves postpartum [8]. Although it rarely recurs in subsequent pregnancy, recurrence was observed here and hence the permanence of PRCA is not known [3].

## Figures and Tables

**Table 1:**
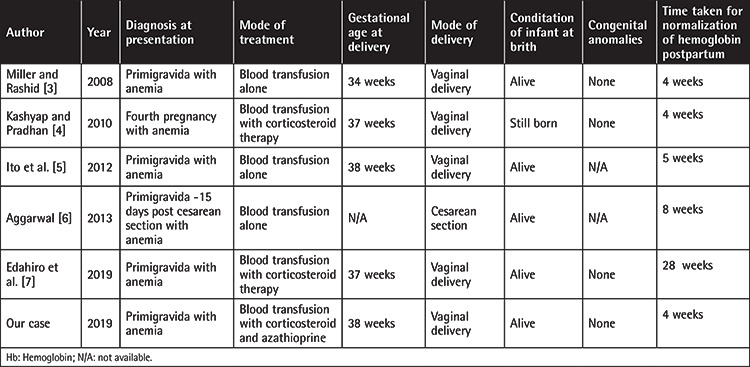
Different modes of treatment used in pregnancy-related pure red cell aplasia by different authors.
